# Broad Dissemination of Plasmids across Groundwater-Fed Rapid Sand Filter Microbiomes

**DOI:** 10.1128/mBio.03068-21

**Published:** 2021-11-30

**Authors:** Rafael Pinilla-Redondo, Asmus Kalckar Olesen, Jakob Russel, Lisbeth Elvira de Vries, Lisbeth Damkjær Christensen, Sanin Musovic, Joseph Nesme, Søren Johannes Sørensen

**Affiliations:** a Section of Microbiology, University of Copenhagen, Copenhagen, Denmark; b Department of Technology, University College Copenhagen, Copenhagen, Denmark; c The Danish Technological Institute, Taastrup, Denmark; CEH–Oxford

**Keywords:** plasmid host range, conjugation, horizontal gene transfer, microbial communities, mobile genetic elements, plasmid, plasmid dissemination, plasmid transfer, rapid sand filters

## Abstract

Biological rapid sand filtration is a commonly employed method for the removal of organic and inorganic impurities in water which relies on the degradative properties of microorganisms for the removal of diverse contaminants, but their bioremediation capabilities vary greatly across waterworks. Bioaugmentation efforts with degradation-proficient bacteria have proven difficult due to the inability of the exogenous microbes to stably colonize the sand filters. Plasmids are extrachromosomal DNA elements that can often transfer between bacteria and facilitate the flow of genetic information across microbiomes, yet their ability to spread within rapid sand filters has remained unknown. Here, we examine the permissiveness of rapid sand filter communities toward four environmentally transmissible plasmids, RP4, RSF1010, pKJK5, and TOL (pWWO), using a dual-fluorescence bioreporter platform combined with fluorescence-activated cell sorting (FACS) and 16S rRNA gene amplicon sequencing. Our results reveal that plasmids can transfer at high frequencies and across distantly related taxa from rapid sand filter communities, emphasizing their potential suitability for introducing bioremediation determinants in the microbiomes of underperforming water purification plants.

## INTRODUCTION

Safe drinking water is becoming a scarce commodity in many parts of the world, and several countries rely on groundwater systems for its supply (1). However, with the advent of modern agriculture, urbanization, and other anthropogenic practices, groundwater reservoirs are threatened by the leaching of chemical pollutants and their toxic degradation products (2). For example, pesticide contamination has been reported to be widespread and recalcitrant among subsoil aquifers, thus constituting an increasing environmental and human health concern (3, 4).

Biological rapid sand filters (sand filters) are commonly employed for the treatment of raw groundwater. Apart from efficiently removing large, suspended particles, and other impurities, sand filters are involved in the biodegradation of organic matter and ammonium removal, processes that heavily rely on the resident bacterial communities (5). Importantly, this water purification approach constitutes a relatively cost-effective and environmentally friendly practice, in contrast to other more advanced technologies, including reverse osmosis (6), advanced oxidation (7), and granular activated carbon (8). Given that the microbial communities in sand filters are not naturally adapted for the removal of anthropogenic compounds, groundwater contaminants often filter though unaltered into the drinking water. To mitigate this problem, the amendment of sand filters with bacteria harboring the desired catabolic genes has been proposed as a means of enhancing the degrading potential of underperforming waterworks (9). Nonetheless, such bioaugmentation strategies have met little success because of the low retention times of the introduced strains, a problem that is attributed mainly to the colonization resistance (biological barrier effect) exerted by the indigenous sand filter microbial communities (9, 10).

While the genes involved in the degradation of xenobiotic compounds can sometimes be borne on the chromosomes of bacteria, they are often found to be carried naturally by diverse mobile genetic elements (MGEs) (11–13). Among these, conjugative and mobilizable plasmids are of particular interest since they are widely recognized as effective vectors for the dissemination of genetic traits across microbiomes (14). Consequently, the delivery of plasmids harboring desired catabolic gene cargos presents itself as a promising alternative to strain-based bioaugmentation strategies. Since the establishment of a plasmid donor strain within a microbial community is not a prerequisite for transfer (15), this approach might serve as a “Trojan horse strategy” to enhance the degrading capabilities of sand filters while bypassing the aforementioned colonization resistance hurdle. Indeed, the use of transmissible plasmids as vehicles to manipulate or enhance complex microbial communities *in situ* has gained traction in recent years, with special focus on engineering the mammalian gut microbiome (16, 17). Although the potential of plasmid-derived bioremediation approaches has indeed been contemplated (18), an adequate understanding of the permissiveness of bacterial communities to the dissemination of exogenous plasmids is currently lacking.

Traditional approaches employed for studying plasmid-mediated horizontal gene transfer present several caveats. While classical experimental setups have been limited primarily to investigating transfer within genetically homogeneous populations or between prototypical laboratory strains, bioinformatics-based comparative genomic analyses are inherently biased toward capturing only those transfers that have become stable throughout evolutionary timescales (19). Furthermore, although metagenomic analyses can indeed reveal the presence of natural plasmids across microbiomes, inferring information about their host range and transfer kinetics is currently unattainable. Thus, to address these challenges, more relevant high-throughput experimental approaches which monitor the spread of fluorescently tagged plasmids within bacterial communities via fluorescence-activated cell sorting (FACS) have been developed (19). These setups have ushered in a better understanding of the natural transfer frequencies and taxonomic dissemination networks of plasmids across diverse environments, including soil and wastewater ecosystems (20). It is noteworthy that these studies have shed light on the remarkably promiscuous nature of certain plasmids, shown to readily transfer into an extremely wide range of phylogenetically distant taxa (21–23).

**FIG 1:**
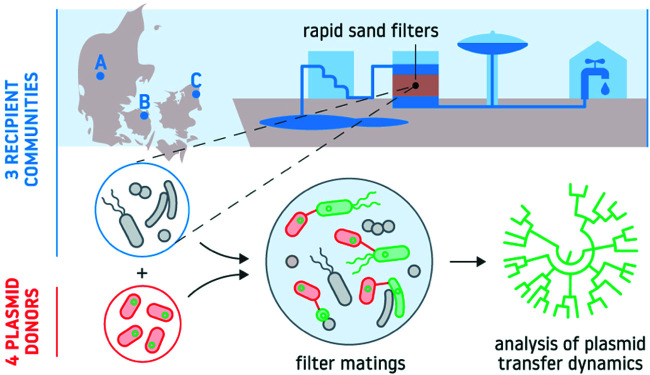
Schematic of the experimental setup. Filter matings were carried out by challenging three sand filter recipient communities (extracted from waterworks in Kerteminde, Bregnerød, and Herning) with four plasmid-donor strain combinations independently (P. putida carrying either pKJK5, TOL, RSF1010, or RP4). Plasmid transfer dynamics (transfer frequency and host range) were monitored using a dual-fluorescence bioreporter platform in combination with high-throughput FACS and 16S rRNA gene amplicon sequencing of transconjugant and recipient cells.

Here, we investigate the potential permissiveness of bacterial communities originating from sand filters of water purification plants from three different geographic locations in Denmark (Kerteminde, Herning, and Bregnerød) to the transfer of four fluorescently tagged environmental plasmids (pKJK5, TOL, RSF1010, and RP4) (Fig. 1). To address this question, we challenged the recipient sand filter communities with each plasmid using Pseudomonas putida as the plasmid donor strain. Plasmid transfer was monitored utilizing a well-established dual-fluorescence bioreporter platform in combination with high-throughput FACS and 16S rRNA gene amplicon sequencing of transconjugant and recipient cells (20). We report the first estimates of plasmid dissemination frequencies and host ranges within sand filter microbiomes, revealing high plasmid transfer frequencies and broad dissemination across bacterial taxa. Taken together, our data demonstrate the potential biotechnological application of natural plasmids for delivering desired genetic determinants among microbial sand filter communities.

## RESULTS AND DISCUSSION

### Transfer efficiencies vary across sand filter recipient communities and plasmid-donor strain combinations.

As a first step to evaluate the feasibility of plasmids for the potential spread of exogenous genes among sand filter microbiomes, we explored the potential uptake of plasmids by bacterial communities originating from this environment. For this purpose, we conducted meta-parental matings in which bacteria extracted from 3 different waterworks in Denmark (Kerteminde, Herning, Bregnerød) (see Fig. S1 in the supplemental material) were challenged with a donor strain carrying one of the following green fluorescent protein (GFP)-tagged plasmids: pKJK5, RP4, RSF1010, or TOL. In order to best assess the possibility of transfer, special attention was given to the choice of a relevant plasmid donor strain and plasmids. Pseudomonas putida was selected as the plasmid donor model organism because it is a common dweller of water and soil environments (24). Four different plasmids were chosen on the basis of (i) their intrinsic horizontal transferability properties, by being either conjugative (self-transmissible) or mobilizable (non-self-transmissible yet able to use the conjugative machinery of a co-occurring conjugative plasmid for transfer), and (ii) their ability to replicate in hosts that naturally feed into drinking water reservoirs (e.g., Pseudomonas) (Table 1).

**TABLE 1:** Plasmids used in this study^*a*^

Plasmid	Inc family	Genotypic and phenotypic characteristics	Size (kb)	Transfer	Reference
pKJK5	IncP-1ε	*P*_A1-O4/O3_::*gfp* Tmp^r^ Tet^r^	54	Conjugative	21
RP4	IncP-1α	*P*_A1-O4/O3_::*gfp* Amp^r^ Km^r^ Tet^r^	60	Conjugative	50
TOL/pWW0	IncP-9	*P*_A1-O4/O3_::*gfp* Km^r^	117	Conjugative	83
RSF1010	IncP-4 (IncQ)	*P*_A1-O4/O3_::*gfp* Km^r^ Strep^r^	8.7	Mobilizable	39

a The bacterial strain used was P. putida KT2440 (*lacI*^q^-*Plpp*-*mCherry* Km^r^) (21). Tmp^r^, trimethoprim resistance; Tet^r^, tetracycline resistance; Amp^r^, ampicillin resistance; Km^r^, kanamycin resistance; Strep^r^, streptomycin resistance; *gfp*, green fluorescent protein gene.

10.1128/mBio.03068-21.1 FIG S1 Sand filter sampling locations. Google map caption indicating the locations of the waterworks in Denmark from which sand filter samples were taken: Herning (A), Kerteminde (B), and Bregnerød (C). Imagery © 2021 TerraMetrics, Map data © 2021 GeoBasis-DE/BKG (© 2009), Google. Download FIG S1, PDF file, 2.2 MB. Copyright © 2021 Pinilla-Redondo et al. 2021 Pinilla-Redondo et al. https://creativecommons.org/licenses/by/4.0/ This content is distributed under the terms of the Creative Commons Attribution 4.0 International license.

**FIG 2:**
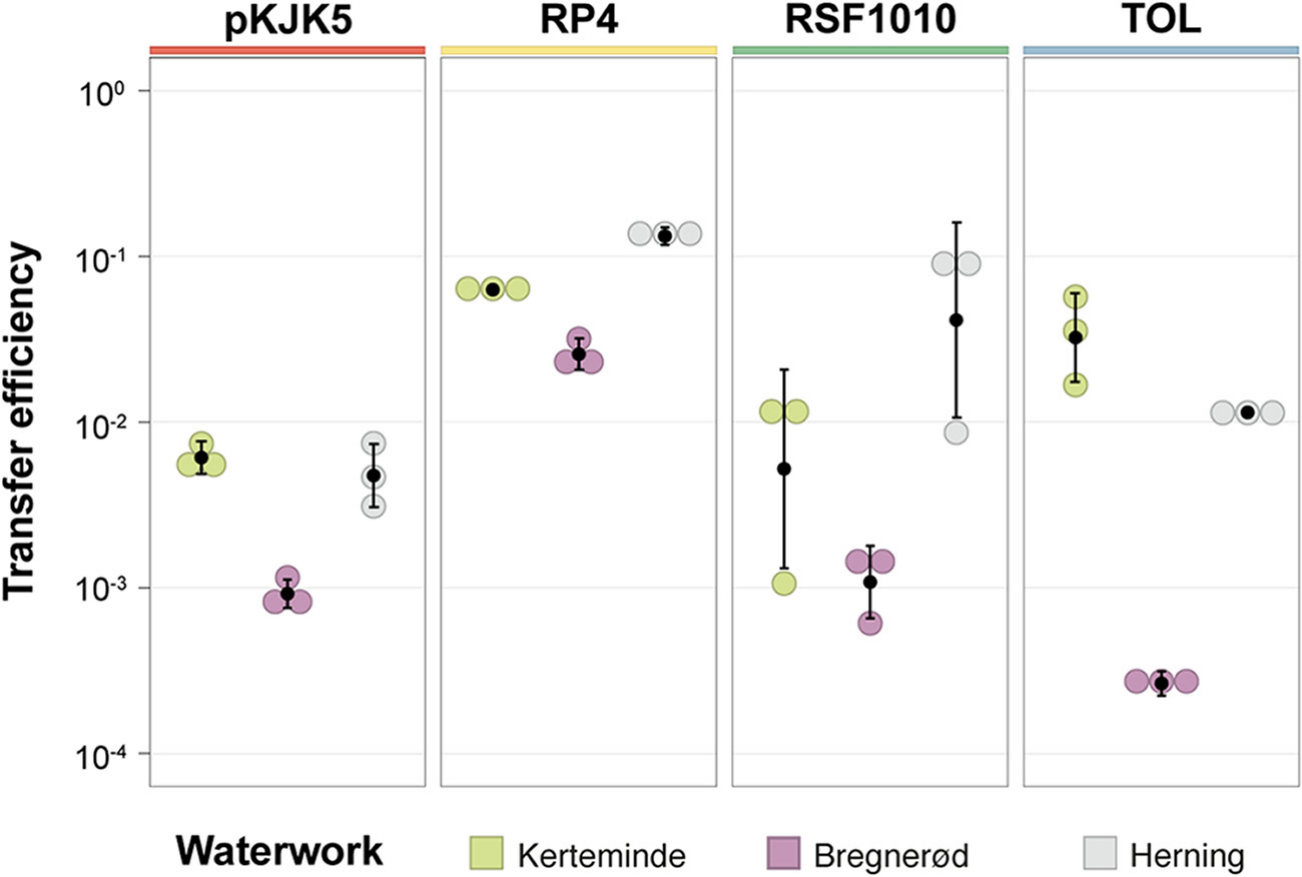
The efficiencies of plasmid transfer vary across rapid sand filter communities and plasmid-donor combinations. Transfer efficiencies of pKJK5, RP4, RSF1010, and TOL resulting from filter matings using P. putida as the plasmid donor and recipient communities originating from the sand filters of the waterworks in Kerteminde, Herning, and Bregnerød, displayed by color. Transfer efficiencies are expressed as the number of transconjugants divided by the geometric mean of the numbers of donor and recipient cells, for each mating outcome. Error bars indicate the standard deviations of the means from three independent filter mating replicates; the black dot represents the mean (see Table S1 in the supplemental material).

The mean transfer efficiencies observed for the tested plasmids ranged from 10^−4^ to 10^−1^ across sand filter communities and plasmid-donor strain combinations (Fig. 2), indicating that bacteria originating from these environments are permissive to the uptake of exogenous plasmids. Overall, the four plasmids showed differences in their transfer efficiencies between sand filters, implying the existence of specific plasmid transfer bottlenecks across recipient communities. Interestingly, Bregnerød recipients exhibited lower transfer efficiencies than Herning and Kerteminde for all tested plasmid-donor combinations, and RP4 showed more consistently high transfer efficiencies across sand filter communities (Fig. 2). These results may reflect differences in the availability of suitable donor-recipient encounters and/or nuances in compatibility between plasmids and the genomic backgrounds that they sample across the different sand filter bacterial communities. A plasmid’s entry and stable maintenance within a new host can be influenced by the host’s innate and adaptive barriers against incoming foreign DNA (25, 26) and conflicts with coresident MGEs. While the former includes bacterial defense systems, such as restriction modification (27), CRISPR-Cas (28), and Wadjet (29), the latter involves plasmid incompatibility issues with indigenous plasmids (30) or may result from plasmid-plasmid competition dynamics, such as those enforced by entry exclusion systems (31, 32) or plasmid-encoded CRISPR-Cas systems (33, 34).

10.1128/mBio.03068-21.7 TABLE S1 Flow cytometry cell counts and transfer efficiency calculations. Download Table S1, XLSX file, 0.01 MB. Copyright © 2021 Pinilla-Redondo et al. 2021 Pinilla-Redondo et al. https://creativecommons.org/licenses/by/4.0/ This content is distributed under the terms of the Creative Commons Attribution 4.0 International license.

Although the TOL plasmid revealed comparatively low transfer frequencies within Bregnerød sand filter communities (<10^−3^), its transfer within Kerteminde and Herning sand filter communities was relatively high (between 10^−2^ and 10^−1^). Notably, this plasmid is of particular interest in the context of groundwater bioremediation because it naturally carries genes encoding enzymes involved in the catabolism of toluene and xylenes, which confer on its bacterial hosts the ability to degrade several pesticides (35). Moreover, RP4 and pKJK5 belong to the IncP1 group of conjugative plasmids, a diverse family in which some members are also known to harbor pesticide-degrading genes (12, 13, 35). Unlike in previous work (23), pKJK5 showed lower transfer frequencies than RP4 (∼1 order of magnitude difference) across all water work microbial communities, emphasizing the importance of studying plasmid transfer in a case-by-case manner and of striving to address transfer dynamics in more relevant spatiotemporal settings.

The high transfer efficiencies of RP4 and pKJK5 across all three waterworks correspond with results of other studies revealing high transfer rates of IncP1 plasmids in soil (21) and wastewater (23, 36). While RP4, pKJK5, and TOL are self-transmissible, RSF1010 does not contain the complete set of necessary genes for conjugation. As such, RSF1010 can transfer into recipient cells only by borrowing components of the conjugative machinery from co-occurring conjugative elements (e.g., integrative conjugative elements [ICEs] and conjugative plasmids) through processes known as mobilization and retromobilization (37, 38). Therefore, monitoring the dissemination of a mobilizable plasmid presents a unique opportunity to measure the intrinsic ability of sand filter communities to mobilize non-self-transmissible plasmids.

Congruently with previous findings, RSF1010 transfer was in the range of 1 order of magnitude lower than that of RP4 (39), except within the recipient microbial community originating from Herning (Fig. 2). The ability of RSF1010 to transfer at high frequencies is remarkable considering its dependency on the availability of compatible conjugation machinery in *trans*. These data indicate a high prevalence of naturally occurring conjugative elements, such as plasmids, in sand filter communities and demonstrate that plasmid mobilization is an effective gene delivery mode in this environment. The plasmids responsible for mobilizing RSF1010 are likely not part of the IncP-1 family, as the resultant incompatibility and entry exclusion dynamics would have prevented the high transfer frequency observed for RP4 or pKJK5 in the communities. Instead, members of the IncW group may participate in the mobilization of RSF1010, as was shown previously (40).

Because the conjugative transfer machinery often occupies a large fraction of a plasmid’s genome (14), mobilizable plasmids may allow for a larger proportion of accessory gene cargos, making them particularly suitable vectors for spreading multiple pesticide-degrading genes. Moreover, since mobilizable plasmids tend to exist in high copy numbers (41), the increased gene dosage might lead to higher expression levels of bioremediation determinants. Interestingly, Herning sand filter communities showed a higher mobilizing potential than those of Kerteminde and Bregnerød, suggesting that the presence of compatible conjugative elements may be variable across sand filters. Together, these results highlight the relevance of designing parallel meta-mobilome studies to investigate the indigenous pool of MGEs (42, 43). Such studies would enable a deeper understanding of the factors affecting the plasmid transfer potential of exogenous plasmids within complex microbial communities.

### Plasmid transfer host ranges across sand filter recipient communities.

Although transfer frequency measures provide valuable knowledge regarding the quantitative dissemination potential of plasmids within a given microbial community, they do not inform about the taxonomic permissiveness of communities toward incoming plasmids. In order to explore the transfer host range of the four plasmids across sand filter communities, we isolated the transconjugant populations via cell sorting (FACS) after the populations mated and characterized them by 16S rRNA gene amplicon sequencing.

**FIG 3:**
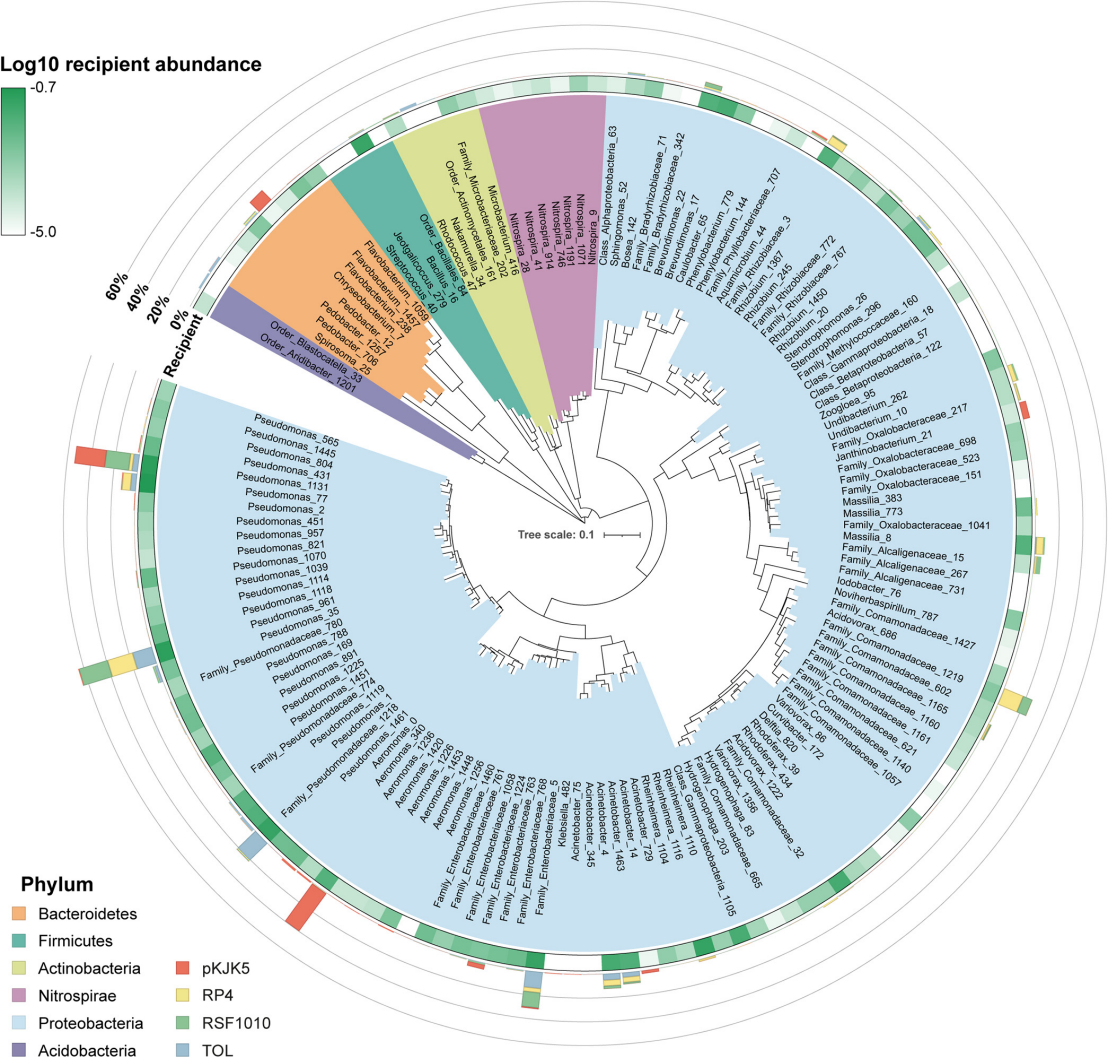
Taxonomic compositions of the transconjugant pools across plasmid-donor combinations. Phylogenetic tree showing the identified transconjugant OTUs across all filter matings using P. putida as the plasmid donor strain. Only OTUs detected in all 3 replicates of at least one sample group are displayed. Background colors radiating from the tree indicate the different phyla to which transconjugants belong, as indicated in the key. The abundance of each OTU in the different transconjugant pools is represented by a stacked bar plot in the outer concentric lane, color-coded according to the four plasmid-donor combinations (TOL, RP4, pKJK5, and RSF1010). The relative abundances (log_10_ transformed) of transconjugant OTUs in the rapid sand filter recipient communities (recipients mated alone and sorted), labeled “Recipient,” are displayed in the innermost lane (green).

**FIG 4:**
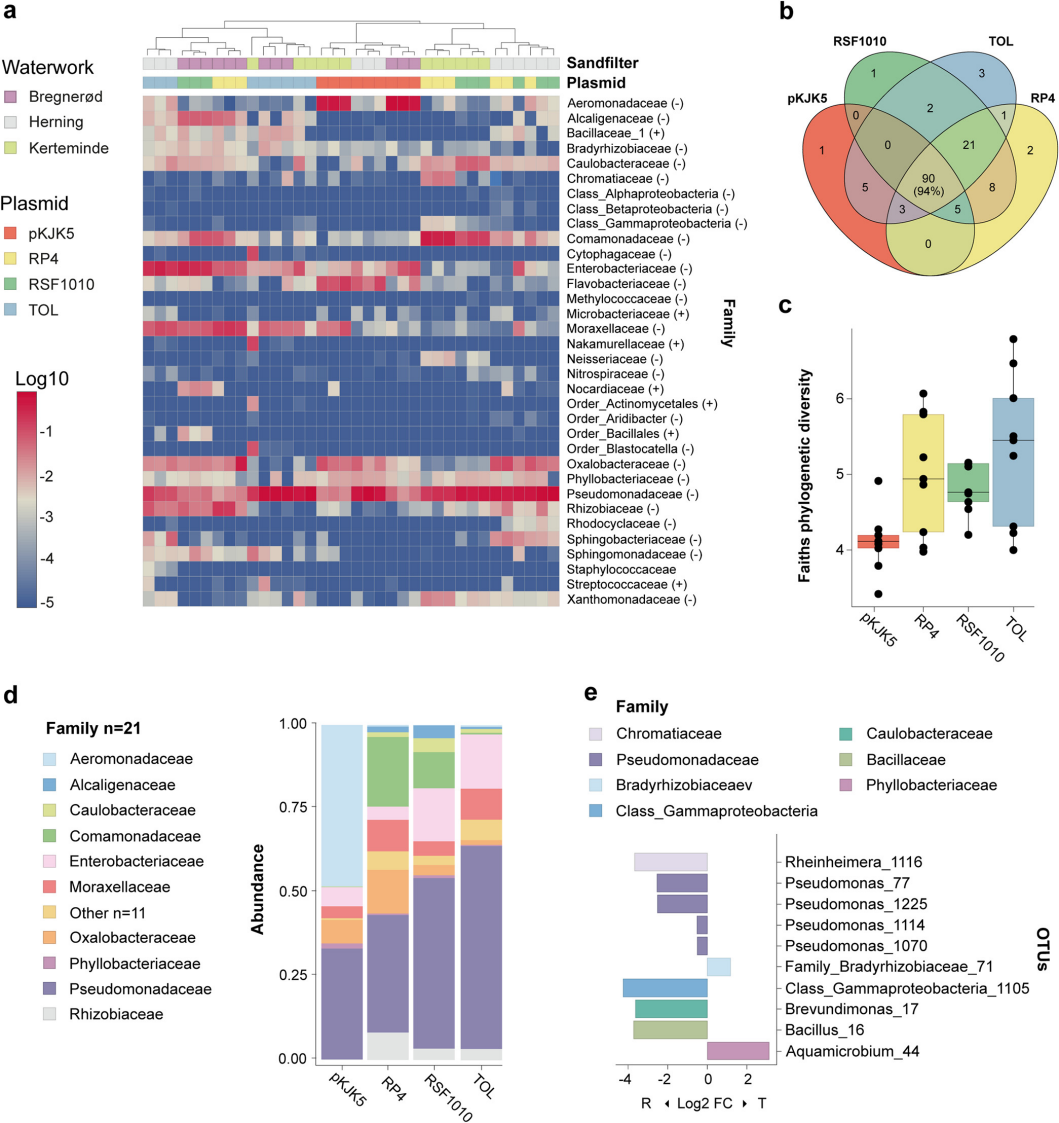
Analysis of the identified transconjugant pools. (a) Heatmap representing the log_10_ relative abundances of the bacterial families identified across transconjugant pools. The dendrogram shows clustering of samples according to taxonomic abundance, using the Ward method (81). Filter mating samples are color-coded according to the waterwork sand filter community and plasmid used, as indicated in the figure legend (top left). (b) Venn diagram displaying the distribution of shared OTUs and the relative abundances of the 90 OTUs shared across all sorted transconjugant populations. (c) Boxplot showing Faith's phylogenetic diversity measure (71) for the 4 plasmid-specific transconjugant pools. (d) Relative abundance distribution of the 90 OTUs (21 families) shared across all transconjugant pools (see panel b). The top 10 most abundant families are shown, and the rest are grouped under “Other.” (e) Bar plot showing the log_2_ fold change in abundance between the FACS-sorted recipient and transconjugant OTU pools. Only the OTUs which revealed both a significant abundance change (Wilcoxon test with false discovery rate [82], adjusted *P* < 0.05) and an absolute log_2_ fold change above 0.5 (representing 10 OTUs out of the total 142) are displayed. The log_2_ fold change in relative abundance for each plasmid can be found in Fig. S5.

Out of all filter mating combinations, a total of 142 distinct transconjugant operational taxonomic units (OTUs) were detected through 16S rRNA sequencing (Fig. 3). The transconjugant fractions consisted largely of taxa from the phylum *Proteobacteria*, with high abundances of members from the *Pseudomonadaceae*, *Aeromonadaceae*, and *Enterobacteriaceae* families (Fig. 3 and 4a), consistent with previous reports of the host ranges of the 4 plasmids tested (21, 23, 36, 44). Interestingly, plasmid transfer was observed in members of the Gram-positive phyla *Actinobacteria* and *Firmicutes* (Fig. 3), highlighting the exceptional ability of certain MGEs to cross distant phylogenetic barriers (45). In agreement with previous studies, *trans*-Gram transfer appeared to comprise only a small fraction of detected transfer events (21, 23, 36, 46) (Fig. 3; Fig. S4). Future studies are needed to investigate the extent to which these plasmids can be stably maintained within Gram-positive hosts or whether these bacteria may comprise replicative dead ends for these MGEs.

10.1128/mBio.03068-21.4 FIG S4 Phylogenetic composition of the samples analyzed in this study. Bar plots show the relative abundances of taxa at the phylum (top) and family (bottom) levels for all sequenced samples, i.e., original sand filter communities (t0; average from 4 replicates), FACS-sorted recipients (filter; average from 3 replicates), and transconjugant pools (plasmid; average from 3 replicates), grouped by sand filter water work location and plasmid-donor combination. Taxa below 0.1% relative abundance for the phylum level and 1% for the family level have been grouped into “other.” Raw data are found in Table S2 in the supplemental material. Download FIG S4, PDF file, 0.2 MB. Copyright © 2021 Pinilla-Redondo et al. 2021 Pinilla-Redondo et al. https://creativecommons.org/licenses/by/4.0/ This content is distributed under the terms of the Creative Commons Attribution 4.0 International license.

10.1128/mBio.03068-21.5 FIG S5 Log_2_ fold abundance changes between the FACS-sorted recipient and transconjugant OTU pools, broken down by plasmid. The transconjugant pools for each plasmid were analyzed separately. Only OTUs which displayed a significant abundance change using the Wilcoxon test adjusted for multiple testing (adjusted *P* < 0.05) are displayed. Furthermore, only OTUs showing a fold change greater than 0.5 log_2_ are shown (i.e., increased in the transconjugant pool, compared to the recipient; 10 out of the total 147 transconjugant OTUs). Download FIG S5, PDF file, 0.2 MB. Copyright © 2021 Pinilla-Redondo et al. 2021 Pinilla-Redondo et al. https://creativecommons.org/licenses/by/4.0/ This content is distributed under the terms of the Creative Commons Attribution 4.0 International license.

It is noteworthy that 90 distinct OTUs pertaining to 21 different families were detected across all plasmid-donor combinations and comprised 94% of the overall transconjugant pools (Fig. 4b and d). These results reinforce the notion that microbial communities harbor a core superpermissive fraction of bacteria that more readily engage in the uptake (and potential retransfer) of incoming MGEs (21, 23). In accordance with previous findings (21, 23, 36, 46, 47), the core permissive taxa consisted mainly of different *Proteobacteria*, including genera such Pseudomonas, Acinetobacter, *Aeromonas*, and members of the *Enterobacteriaceae* and *Rhizobiaceae* families (Fig. 3, heat map lane, and Fig. 4a and d). These results suggest common strategies in the promiscuity of certain taxa toward foreign incoming DNA.

Faith’s phylogenetic diversity index was evaluated across plasmid-specific transconjugant pools as a proxy for taxonomic breadth of transfer (Fig. 4c). Overall, our analysis revealed similar transfer ranges for the different plasmids under the conditions tested, except for pKJK5, which appeared to show a comparatively low transconjugant phylogenetic breadth (Fig. 4b and c), significantly lower than that of the TOL and RP4 plasmids (Tukey’s honestly significant difference test, adjusted *P*  < 0.05). Interestingly, the plasmid RSF1010 displayed a relatively broad transfer host range (Fig. 4c), transferring into 127 distinct OTUs despite being non-self-transmissible (Fig. 4b). Our results thus support the idea that because mobilizable plasmids can often be shuttled by diverse type IV secretion system machineries, they can disseminate remarkably across microbial communities. Furthermore, it has been shown that RSF1010 can be maintained stably in Gram-positive bacteria, such as in members of the *Actinobacteria* (48). Notably, these exceptional properties have been proposed to allow mobilizable plasmids access to even broader taxonomic host ranges than self-transmissible plasmids under natural conditions (37, 39).

Importantly, certain taxa that were represented in the filter-mated recipient communities at low abundances were found to be relatively enriched in the corresponding transconjugant pools, indicating their high permissiveness toward incoming plasmids. These include members of the genera *Aquamicrobium* and the family *Bradyrhizobiaceae*. On the other hand, certain abundant recipient genera, such as *Brevundimonas* and *Bacillus*, and certain Pseudomonas species were poorly represented in the transconjugant pools (Fig. 4e; Fig. S4).

All together, these results emphasize the intricate dynamics surrounding plasmid-host interactions and the need for a deeper characterization of the recipient and transconjugant pools. Given that both innate and adaptive barriers against foreign invading genetic elements are extremely diverse and heterogeneously spread across bacterial taxa (25), differences in plasmid transfer between communities and community members (even among closely related taxa) are expected. Future studies will benefit from the advent of complementary procedures to 16S rRNA gene amplicon sequencing, such as advances in sequencing single-cell amplified genomes (SAGs) (49). Indeed, comparative genomic analyses of recipient and transconjugant cell SAG data may provide insights into the genetic factors influencing the promiscuous or refractory nature of bacteria toward incoming plasmids.

A clear distance between the indigenous sand filter communities (here called T0) and the recipient communities from the filter matings was observed (Fig. S6, permutational multivariate analysis of variance [PERMANOVA] performed on weighted UniFrac distances, *R*^2^ = 0.71, *P* = 0.001). This is likely due to a large fraction of the total indigenous bacteria from the sand filters not being able to grow under the conditions tested. Additionally, since it is expected that certain transconjugant cells may miss detection due to rapid plasmid loss or inadequate GFP expression levels, it is likely that the transfer host ranges of these plasmids are even broader than reported here. Such prospective limitations, however, are not necessarily inconsequential, as ecologically and evolutionarily important transfer events can indeed be short-lived. These considerations serve as a reminder that the paradigms derived from our model system may not faithfully extend to the natural environment, where the microbial taxonomic diversity and physicochemical conditions are significantly different. However, given that frequent and broad-range transfer was detected within the cultivable fraction of all the sand filter communities investigated here (Fig. 2 and 3), we conclude that rapid sand filters likely constitute environments with high permissibility potentials toward incoming plasmids.

10.1128/mBio.03068-21.6 FIG S6 Culturing rapid sand filter bacteria substantially affects the original microbial community composition. Principal-coordinate analysis (PCoA) of the weighted UniFrac distances between the recipient sand filter communities, as indicated by icon shape, and the recipient communities (the original sand community [T0; blue] and those sorted post-filter mating [Filter; red]). Download FIG S6, PDF file, 0.07 MB. Copyright © 2021 Pinilla-Redondo et al. 2021 Pinilla-Redondo et al. https://creativecommons.org/licenses/by/4.0/ This content is distributed under the terms of the Creative Commons Attribution 4.0 International license.

The contamination of groundwater ecosystems with anthropogenic pollutants (e.g., pesticides) has severe environmental repercussions that challenge the production of potable water. Notably, many groundwater-fed water works are not equipped with the natural bioremediation capabilities required to face this growing concern. Cell-based bioaugmentation practices have been proposed to mitigate this problem, yet these efforts are typically limited due to out-competition of the inoculated strains by indigenous microbes (ecological barrier effect). On the other hand, plasmid-based bioaugmentation approaches may have the potential to enhance the degradative competence of the already established, ecologically competitive, autochthonous microbial communities.

This study revealed the significant ability of natural plasmids to transfer at high frequencies and across distantly related taxa within groundwater-fed rapid sand filter communities in the absence of plasmid selection, indicating their potential suitability as vectors for the spread of bioremediation determinants in water purification plants. Furthermore, our data show that mobilizable plasmids, despite being non-self-transmissible, can disseminate widely, comparably to certain broad-host-range conjugative plasmids. Future work is required to assess the biotechnological applicability and long-term maintenance of exogenous plasmids within sand filter communities.

## MATERIALS AND METHODS

### Strains, plasmids, and sand filter recipient communities.

The bacterial strains and plasmids and their relevant characteristics are listed in Table 1. Pseudomonas putida KT2440 (chromosomally tagged by *lacI*^q^*-Plpp-mCherry*), carrying either pKJK5, RP4, TOL, or RSF1010, was used as the plasmid donor strain in the mating experiments. Sand filter sediments were sampled from water purification plants in Denmark: Kerteminde (55°25′47.5″N, 10°38′18.2″E), Herning (56°08′48.5″N, 8°56′33.7″E), and Bregnerød (55°48′51.9″N, 12°22′36.2″E), representing geographically distinct rapid sand filter microbial communities (Fig. S1). The indigenous sand filter bacteria (T0) were extracted using the Nycodenz gradient extraction method (50). Briefly, the sand material was ground with a mortar in 50 mM TTSP (tetrasodium pyrophosphate buffer) and layered on top of the Nycodenz solution (Nycomed Pharma, Norway; 1.3 g/mL) prior to centrifugation (8,500 × *g* for 15 min). The upper and intermediate phases containing the bacterial cells were collected, resuspended in 5× volumes of phosphate-buffered saline (PBS), and stored at 5°C until use. The donor strains were routinely grown in LB broth (10 g tryptone, 5 g yeast extract, and 4 g NaCl), using the appropriate antibiotics added at final concentrations of 20 μg/mL for tetracycline and 50 μg/mL for kanamycin. The recipient communities were grown in 5% tryptic soy broth (TSB) overnight at 30°C and 150 rpm to facilitate their recovery from their isolation and cold storage and to enrich for the culturable fraction of bacteria in this environment prior to mating.

### Solid-surface plasmid conjugation assay.

The permissiveness of the sand filter recipient communities toward the exogenous conjugative/mobilizable plasmids was tested using a modified version of a solid-surface meta-parental mating setup described previously (Fig. 1) (21). According to this approach, plasmids are tracked through an inserted *gfp* marker controlled by a *lacI*^q^-repressible promoter. The donor strain additionally harbors a chromosomal *lacI*^q^*-Plpp-mCherry* insertion. Thus, in plasmid donor cells, constitutive LacI production results in repression of the plasmid-encoded GFP, while the constitutive mCherry expression renders the cells red (Fig. 1). The *gfp*-tagged plasmids, however, upon transfer into natural sand filter recipients are able to express GFP because these bacteria lack the *lacI*^q^ insert found in the donor, thus ensuring a green fluorescent phenotype for transconjugant cells (20). We define community permissiveness here as the ability of native bacteria in the recipient rapid sand filter community to receive and express a reporter gene harbored by our plasmids.

Filter matings, as well as negative-control matings with the recipient communities and donor strains grown alone on the filters, were carried out by challenging the extracted sand filter recipient communities with the four plasmid-donor strain combinations independently, in triplicates. Donor and recipient cell suspensions were adjusted to an optical density at 600 nm (OD_600_) of 0.5, and 100 μL of each was mixed at a 1:1 ratio. The resulting suspension was transferred onto sterile 0.2-μm nitrocellulose filters (Advantec) that were placed over 10% TSB agar medium (Sigma-Aldrich) without antibiotic selection. The area of the filter exposed was estimated to be 54 mm^2^, resulting in an initial cell count of approximately 3.6 × 10^5^ cells/mm^2^. When dry, plates were incubated at 30°C for 24 h, and filters were washed with 5 mL PBS to recover cells for FACS analysis (cell counting and sorting). Mating samples were kept at 4°C after recovery from filters and analyzed within a period of 3 to 4 days. The transfer efficiencies were calculated as the number of transconjugant cells (*T*) divided by the geometric mean of the numbers of donor (*D*) and recipient (*R*) cells [$\sqrt{\left(R\times D\right)}$] after mating (51).

### FACS analysis.

Flow cytometric detection of cells was performed by using a FACSAria Illu (Becton Dickson Biosciences, San Jose, CA, USA). The following technical settings were employed. A 70-μm nozzle and sheath fluid pressure of 70 lb/in^2^ were used. GFP was excited by a 488-nm laser (20 mW) and detected on the fluoresceine isothiocyanate A (FITC-A) channel with a bandpass filter of 530/30 nm. mCherry was excited with a 561-nm laser (50 mW) and detected on the phosphatidylethanolamine (PE)-Texas Red-A channel with a bandpass filter of 610/20 nm. Detection thresholds were set to 200 for forward and side scatter (FSC and SSC, respectively). BD FACSDiva software v.6.1.3 was used for operating and analyzing the results.

Bivariate contour plots of particle FSC versus SSC areas were employed to build a gate around the total bacterial population, excluding the background noise. Green and red fluorescent bacterial cells were gated on bivariate contour plots using the area of FITC versus the area of PE-Texas Red. The detection gates used in this study are depicted in Fig. S2. Donor, recipient, and transconjugant counts were made with the “mCherry” (red), “non-red,” and “green-non-red” gates, respectively. Flow cytometric analysis was performed by diluting filter mating samples in PBS to a cell count of 1,000 to 3,000 threshold events/s, processed at flow rate 1. A total of 100,000 bacterial events were recorded for each mating outcome. We sorted cells into 5-mL sterile polypropylene round-bottom tubes (Falcon by Corning, USA) containing 0.5 mL of PBS. Because transconjugant cells often comprise less than 1% of the total cell population, we performed a preliminary sorting round as an enrichment step for transconjugant cells, as described in reference 21. First, 200,000 to 500,000 target transconjugant events were sorted using a flow rate of ∼15,000 events/s and employing the “yield/recovery” settings (both the interrogated drop and the drop adjacent to the target particle are sorted). Subsequently, a second, more restrictive sorting step employing “purity” settings and a threshold rate of <3,000 events/s was carried out to sort high-purity transconjugant cells (any target events falling close to any nontarget events are not sorted). In the second sorting round, 20,000 cells were isolated from all filter mating combinations. Sorted cells were then prepared for subsequent deep amplicon sequencing of 16S rRNA genes.

10.1128/mBio.03068-21.2 FIG S2 Overview of the flow cytometry and FACS gates employed for counting and sorting plasmid-donor, recipient, and transconjugant cells. The different sand filter communities and plasmid-donor strains that were used in this work were grown on solid-surface filters alone and served as negative controls for transfer during filter matings. The resultant log_10_-scaled scatter plots were employed to design an appropriate gating strategy. (i) Bacterial cells (left column, FSC-A versus SSC-A; in blue); (ii) red fluorescent donor cells and non-red recipients (middle column; FSC-A versus PE-Texas Red-A, in red and blue, respectively); (iii) green non-red fluorescent transconjugant cells (right column, FITC-A versus PE-Texas Red-A). The Bregnerød recipient community challenged with P. putida carrying RP4, replicate 2, is displayed in the last row as an example of a filter mating outcome and to show the suitability of the designed transconjugant cell gate. Download FIG S2, PDF file, 0.3 MB. Copyright © 2021 Pinilla-Redondo et al. 2021 Pinilla-Redondo et al. https://creativecommons.org/licenses/by/4.0/ This content is distributed under the terms of the Creative Commons Attribution 4.0 International license.

### Nucleic acid extraction.

Microbial community profiling was carried out by 16S rRNA gene amplicon sequencing for the original sand filter community, T0 (Fig. S3 and S4). DNA was extracted using the NucleoSpin soil kit (Macherey-Nagel) by following the manufacturer’s instructions and by using the lysis buffer SL1 and a bead-beating mechanical lysis step performed on a FastPrep-24 (MP Biomedicals) tissue homogenizer at 6 m/s for 30 s. After filter mating, sorted transconjugant and recipient cells (referred to as “filter”) were pelleted by centrifugation at 10,000 × *g* for 30 min, and cell lysis and DNA extraction were carried out in the thermal cycler by following the protocol detailed by the GenePurgeDirect (NimaGen) direct PCR kit.

10.1128/mBio.03068-21.3 FIG S3 Rarefaction curves of the 16S rRNA gene sequencing profiles for each of the studied samples. Curves show the observed OTUs at 97% similarity in each sample as a function of the number of 16S rRNA gene sequencing reads (*x* axis). Colors indicate the sample groups indicated in the figure legend; recipient communities (original [t0] and sorted postmating [filter]) and transconjugant (sorted postmating [filter]) samples are separated by plasmid (TOL, RSF1010, pKJK5, and RP4) and sand filter location, i.e., Bregnerød (B), Kerteminde (K), and Herning (H). Download FIG S3, PDF file, 0.1 MB. Copyright © 2021 Pinilla-Redondo et al. 2021 Pinilla-Redondo et al. https://creativecommons.org/licenses/by/4.0/ This content is distributed under the terms of the Creative Commons Attribution 4.0 International license.

### 16S rRNA gene amplicon sequencing.

Sequencing libraries were prepared using a dual-PCR setup as described previously (52), targeting variable regions V3 and V4 of the 16S rRNA gene, approximately 460 bp. In the first step, primers Uni341F (5′-CCTAYGGGRBGCASCAG-3′) and Uni806R (5′-GGACTACNNGGGTATCTAAT-3′), originally published by Ye et al. (53) and modified as described in reference 54, were used. In a second PCR step, the primers additionally included Illumina sequence-specific sequencing adapters and a unique combination of indexes for each sample. PCRs were performed in a 25-μL volume using PCRBIO HiFi polymerase and 2 μL template DNA, according to the manufacturers’ instructions and the following program: 95°C for 1 min, followed by 30 or 15 cycles (for, respectively, PCR1 or PCR2) of 95°C for 15 s, 56°C for 15 s, and 72°C for 30 s. After both PCRs, amplicon products were purified using the HighPrep PCR cleanup system (AC-60500; MagBio Genomics Inc., USA) using a 0.65:1 (beads to PCR mixture) volumetric ratio to remove DNA fragments below 100 bp in size. Samples were normalized using a SequalPrep normalization plate (96 wells) kit (Invitrogen, Maryland, MD, USA) and pooled using a 5-μL volume of each. The final pool volume was reduced to concentrate the sequencing library using the DNA Clean and Concentrator-5 kit (Zymo Research, Irvine, CA, USA). The pooled library concentration was determined using the Quant-iT high-sensitivity DNA assay kit (Life Technologies) by following the specifications of the manufacturer. Before library denaturation and sequencing, the final pool concentration is adjusted to 4 nM before library denaturation and loading. Amplicon sequencing was performed on an Illumina MiSeq platform using reagent kit v2 (2 × 250 cycles) (Illumina Inc., CA, US). The MiSeq Controller software Casava 1.8 (Illumina, USA) was used for sequence demultiplexing, and the paired-end FASTQ output files were used for the downstream sequencing analysis. Raw sequence reads were first trimmed of primer sequences used in first PCR using cutadapt (55), and only read pairs for which both primers were found are retained for subsequent analysis. Primer-trimmed sequences are then merged and clustered into OTUs using the UPARSE-OTU algorithm (56) with a 97% pairwise sequence similarity threshold. The taxonomic annotation of each cluster’s representative sequence was performed using mothur (57) using the Ribosomal Database Project database trainset 16 (58; https://www.mothur.org/wiki/RDP_reference_files). An approximate maximum likelihood phylogenetic tree was built with FastTree (59), based on alignment of all reference OTU cluster sequences obtained with mothur align.seqs.

### Sequence and data analyses.

Data analysis was carried out in R (60) through the following R packages: phyloseq (61), reshape2 (62), stringr (63), dplyr (64), and plyr (65). The prevalence method (threshold = 0.25) of the decontam package (66) was used to remove potential contaminants from the data set, removing 3.29% of the total reads. The COEF package (67) was used to remove OTUs that were not present in at least 2 of 3 sample replicates across the whole data set. For the sorted transconjugant samples, a more conservative threshold was applied in order to avoid the conceivably higher influence of contaminant DNA in these lower-biomass samples. Accordingly, OTUs that were not present in all three replicates were removed. Furthermore, OTUs exhibiting a frequency of <10^−4^ in their respective transconjugant pools were not considered in downstream analyses. The T0 samples describing the original sand filter community from Herning were removed from analyses due to indications of a technical error. The ggplot2 (68) and ggpubr (69) packages were used for data visualization, and colors were adjusted using RColorBrewer (70). Faith's phylogenetic diversity metric (71) was calculated with the PhyloMeasures package (72) via the metagMisc package (https://github.com/vmikk/metagMisc).

The statistical software package R (60) was used for analysis of variance (ANOVA) and to calculate Tukey’s honestly significant differences. Weighted UniFrac distances (73) were calculated and plotted with the phyloseq package (61). PERMANOVA tests were done with the vegan package (74), using 999 permutations. Venn diagrams were constructed with the eulerr package (75) through the MicEco package (76). Heatmap plotting was made with the pheatmap package (77), and differential abundance testing analyses were carried out with the DAtest package (78). The taxonomic composition of transconjugant pools across plasmid-donor and sand filter recipient community combinations were made using the iTOL webtool (79), and the phylogenetic tree used as input was written from the phyloseq object using the ape package (80).

### Data availability.

All raw sequence reads data have been deposited in EBI-SRA under BioProject accession number PRJEB36794.

10.1128/mBio.03068-21.8 TABLE S2 16S rRNA gene sequencing data generated in this study. Download Table S2, XLS file, 0.07 MB. Copyright © 2021 Pinilla-Redondo et al. 2021 Pinilla-Redondo et al. https://creativecommons.org/licenses/by/4.0/ This content is distributed under the terms of the Creative Commons Attribution 4.0 International license.
