# Supplementary material for: Broad Dissemination of Plasmids across Groundwater-Fed Rapid Sand Filter Microbiomes
Source: mBio. 2021 Nov 30;12(6):e03068-21. doi: 10.1128/mBio.03068-21 (PMC8630534; doi:10.1128/mBio.03068-21)
Supplement: FIG S1 [file mbio.03068-21-sf001.pdf]

Supplemental Material:

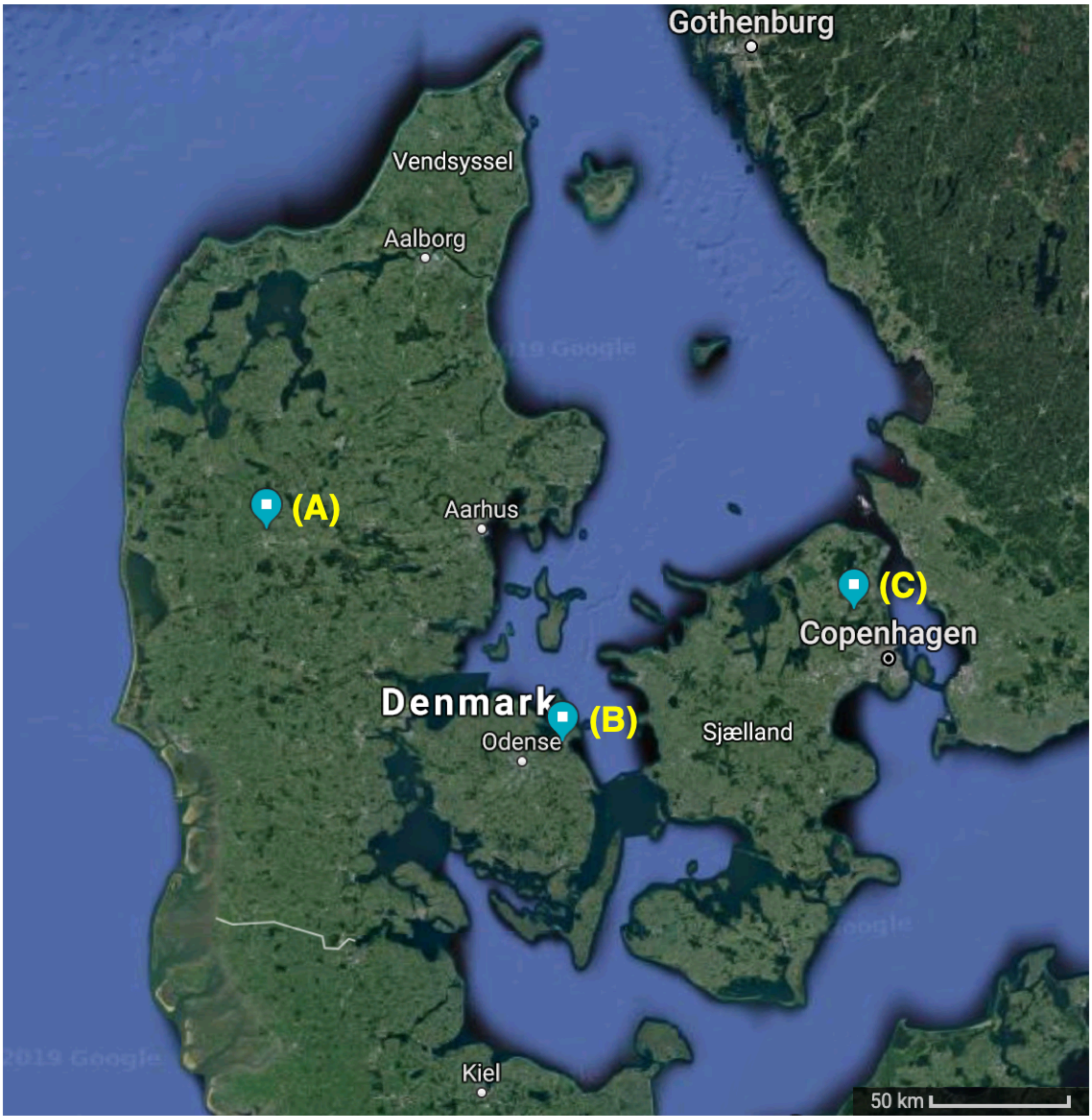

Supplementary Fig. S1. sand filter sampling locations. Google map caption indicating the locations of the waterworks in Denmark from which sand filter samples were taken: (A) Herning; (B) Kerteminde; (C) Bregnerød.
