# Supplementary material for: Broad Dissemination of Plasmids across Groundwater-Fed Rapid Sand Filter Microbiomes
Source: mBio. 2021 Nov 30;12(6):e03068-21. doi: 10.1128/mBio.03068-21 (PMC8630534; doi:10.1128/mBio.03068-21)
Supplement: FIG S4 [file mbio.03068-21-sf004.pdf]

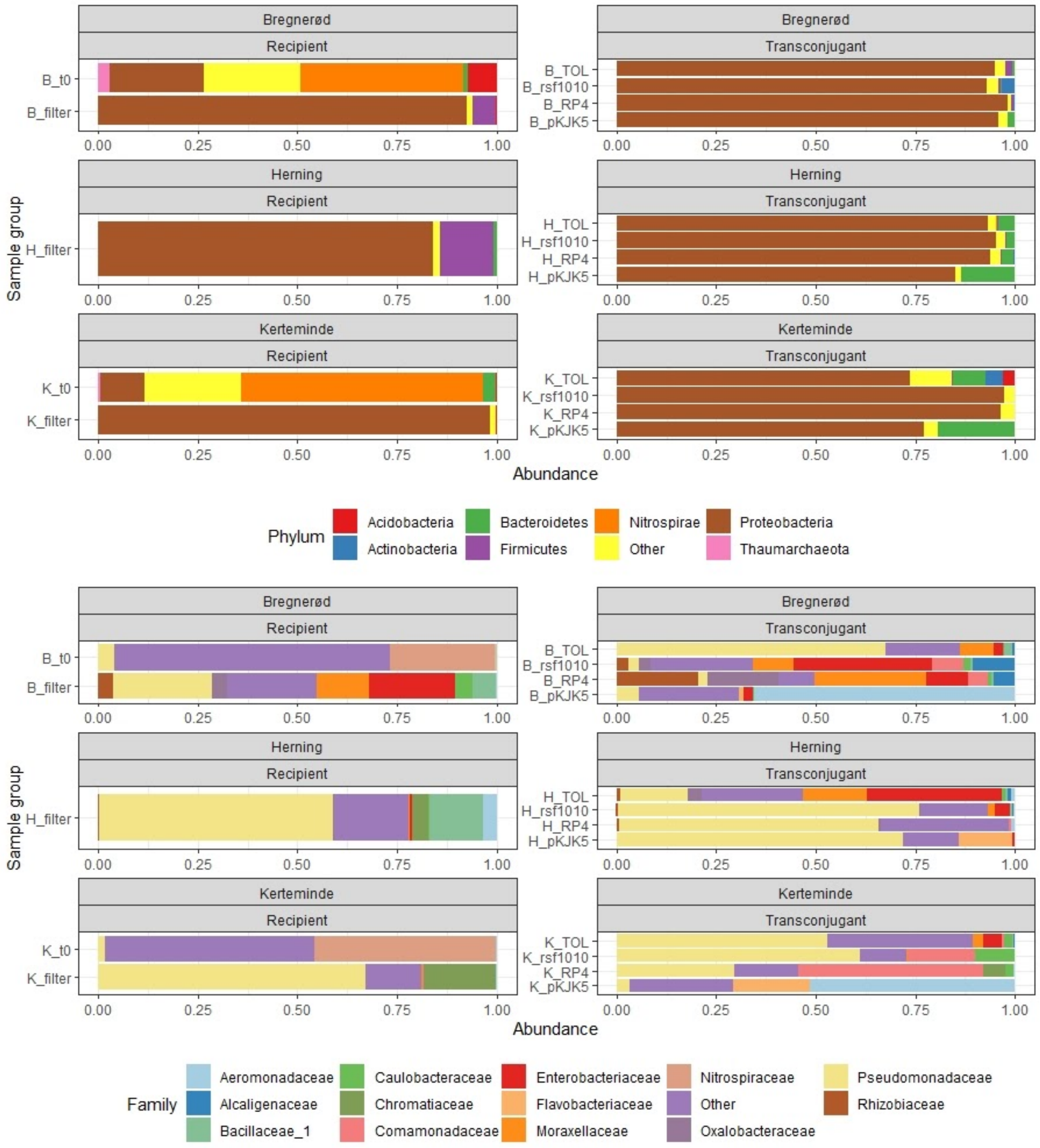

Supplementary Figure S4. Phylogenetic composition of the samples analysed in this study. Bar plots show the relative abundances of taxa at the Phylum (top) and Family (Bottom) levels for all sequenced samples: original sand filter communities ("t0", average of 4 replicates) and FACS-sorted recipients ("Filter", average for 3 replicates) and transconjugant pools ("plasmid", average of 3 replicates), grouped by sand filter water work location and plasmid-donor combination. Taxa below 0.1% relative abundance, for phylum level, and 1% for family level, have been grouped into "other". Raw data found in Supplementary Table S2
