# Supplementary material for: Broad Dissemination of Plasmids across Groundwater-Fed Rapid Sand Filter Microbiomes
Source: mBio. 2021 Nov 30;12(6):e03068-21. doi: 10.1128/mBio.03068-21 (PMC8630534; doi:10.1128/mBio.03068-21)
Supplement: FIG S5 [file mbio.03068-21-sf005.pdf]

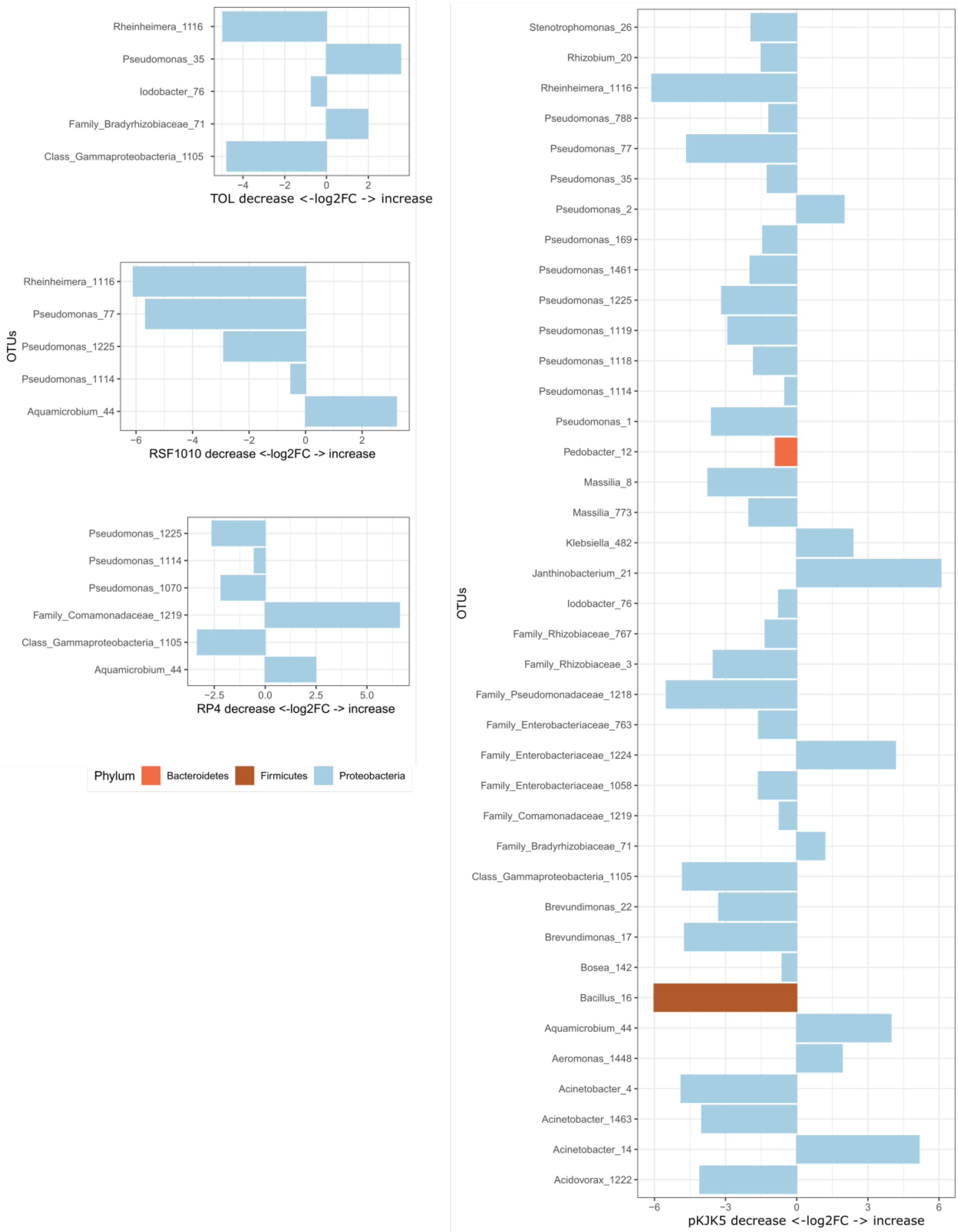

Supplementary Figure S5. Log2 fold abundance changes between the FACS-sorted recipient and transconjugant OTU pools, broken down by plasmid. The transconjugant pools for each plasmid have been analysed separately. Only OTUs which displayed a significant abundance change using the Wilcoxon test adjusted for multiple testing ($p.adj < 0.05$) are displayed. Furthermore, only OTUs showing a fold change greater than 0.5 log 2 are shown (i.e. increased in the transconjugant pool, compared to the recipient); 10 out of the total 147 transconjugant OTUs.
