# Supplementary material for: Broad Dissemination of Plasmids across Groundwater-Fed Rapid Sand Filter Microbiomes
Source: mBio. 2021 Nov 30;12(6):e03068-21. doi: 10.1128/mBio.03068-21 (PMC8630534; doi:10.1128/mBio.03068-21)
Supplement: FIG S6 [file mbio.03068-21-sf006.pdf]

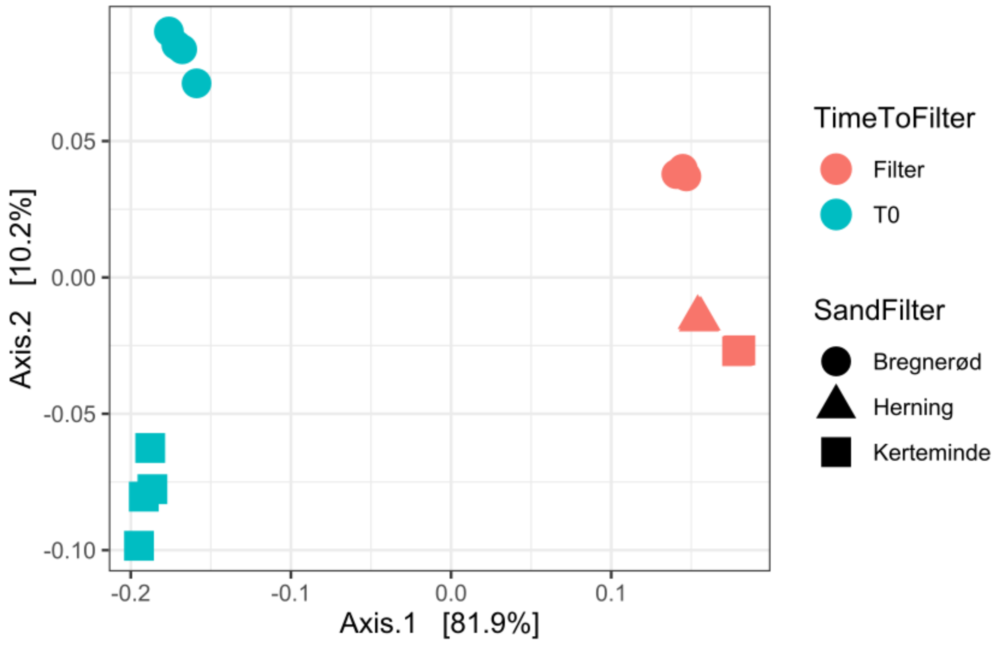

Supplementary Figure S6. Culturing rapid sand filter bacteria substantially affects the original microbial community composition. PCoA of the weighted unifrac distance between the recipient sand filter communities, as indicated by icon shape, and recipient community: original sand community; "T0", and sorted post-filter mating; "Filter", shown in blue and red color, respectively.

Supplementary Table S1. Flow cytometry cell counts and transfer efficiency calculations (Separate Datasheet).

Supplementary Table S2: 16S rRNA gene sequencing data generated in this study (Separate Datasheet).
