# Supplementary material for: Broad Dissemination of Plasmids across Groundwater-Fed Rapid Sand Filter Microbiomes
Source: mBio. 2021 Nov 30;12(6):e03068-21. doi: 10.1128/mBio.03068-21 (PMC8630534; doi:10.1128/mBio.03068-21)
Supplement: FIG S2 [file mbio.03068-21-sf002.pdf]

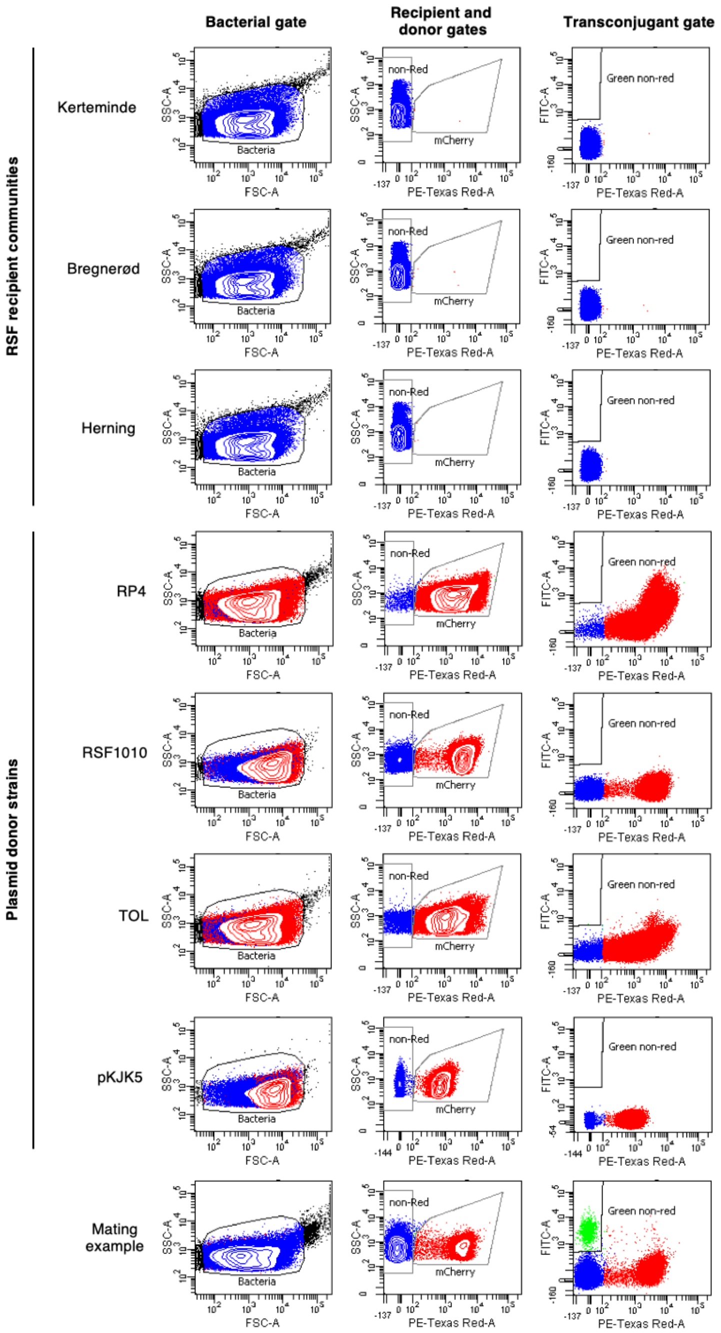

Supplementary Figure S2: Overview of the flow cytometry and FACS gates employed for counting and sorting plasmid-donor, recipient and transconjugant cells. The different sand filter communities and plasmid donor strains that were used in this work were grown on solid-surface filters alone and served as negative controls for transfer during filter matings. The resultant Log(10)-scaled scatter plots were employed to design an appropriate gating strategy; (i) bacterial cells (left column, FSC-A vs. SSC-A; in blue); (ii) red fluorescent donor cells and non-red recipients (middle column; FSC-A vs. PE-Texas-Red-A, in red and blue, respectively); (iii) green non-red fluorescent transconjugant cells (right column, FITC-A vs. PE-Texas-Red-A). Bregnerød recipient community challenged with *P. putida* carrying RP4, replicate 2, is displayed in the last row as an example of a filter mating outcome and to show the suitability of the designed transconjugant cell gate.
