# Supplementary material for: Broad Dissemination of Plasmids across Groundwater-Fed Rapid Sand Filter Microbiomes
Source: mBio. 2021 Nov 30;12(6):e03068-21. doi: 10.1128/mBio.03068-21 (PMC8630534; doi:10.1128/mBio.03068-21)
Supplement: FIG S3 [file mbio.03068-21-sf003.pdf]

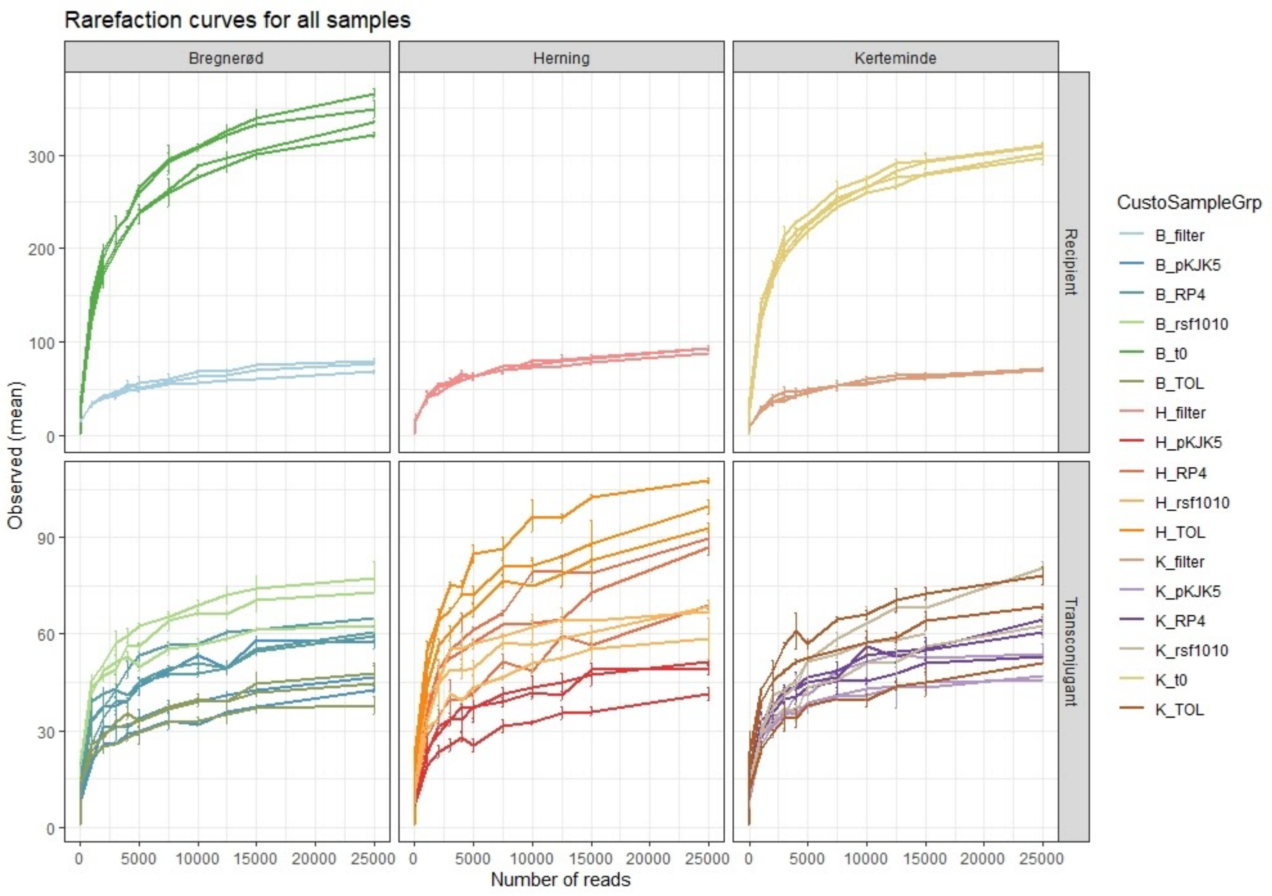

Supplementary Fig. S3. **Rarefaction curves of the 16S rRNA gene sequencing profiles for each of the studied samples.** Curves show the observed OTUs at 97% similarity in each sample as a function of the number of 16S rRNA gene sequencing reads (X-axis). Colors indicate the sample groups indicated in the figure legend; recipient communities (original; “t0”, and sorted post-mating; “filter”) and transconjugant (sorted post-mating; “filter”) samples that are separated by plasmid (TOL, RSF1010, pKJK5, and RP4) and sand filter location: Bregnerød (B), Kerteminde (K), and Herning (H).
